# Stereoselective synthesis of four possible isomers of streptopyrrolidine

**DOI:** 10.3762/bjoc.7.6

**Published:** 2011-01-10

**Authors:** Debendra K Mohapatra, Barla Thirupathi, Pragna P Das, Jhillu S Yadav

**Affiliations:** 1 Division of Organic Chemistry-I, Indian Institute of Chemical Technology (CSIR), Hyderabad-500607, India, Tel/Fax: 0091-40-27193128

**Keywords:** aldol reaction, angiogenesis, cancer, Lewis acid mediated lactamization, streptopyrrolidine

## Abstract

The synthesis of (4*R*,5*R*)-streptopyrrolidine (**1**), (4*S*,5*R*)-streptopyrrolidine (**2**) (4*R,*5*S*)-streptopyrrolidine (**3**) and (4*S,*5*S*)-streptopyrrolidine (**4**) have been achieved in a concise and highly efficient manner via a highly stereoselective aldol type reaction with the trimethylsilyl enolate of ethyl acetate and Lewis acid mediated lactamization as the key reactions in ≈42% yield over six steps starting from D-phenylalanine and L-phenylalanine, respectively. The absolute configuration of the natural product was shown to be (4*S*,5*S*) by comparing its spectral and analytical data with the reported values.

## Introduction

Cancer is at present the second most common cause of death, after cardiovascular diseases, and will become the primary cause in the next 10 to 20 years [1]. Traditional cancer therapies make use of chemotherapy at the maximum tolerated dose. This approach has generally considerable associated toxicity, often with limited success. Therefore, more universal, more effective, and less toxic therapeutic agents are desirable. Recently, inhibition of angiogenesis has been considered as a desirable pathway for preventing tumor growth and metastasis, primarily because of the low potential for toxicity or resistance [2], as well as the potential for treating a broad spectrum of tumor types, arthritis, and psoriasis [3–8]. For this reason, angiogenesis inhibition has become an active area of pharmaceutical research, and over 40 such agents are currently undergoing clinical trials [9]. In particular, efforts have been focused on small-molecules based on inhibitors isolated from natural products that can block tumor angiogenesis [10–11].

**Figure 1:**
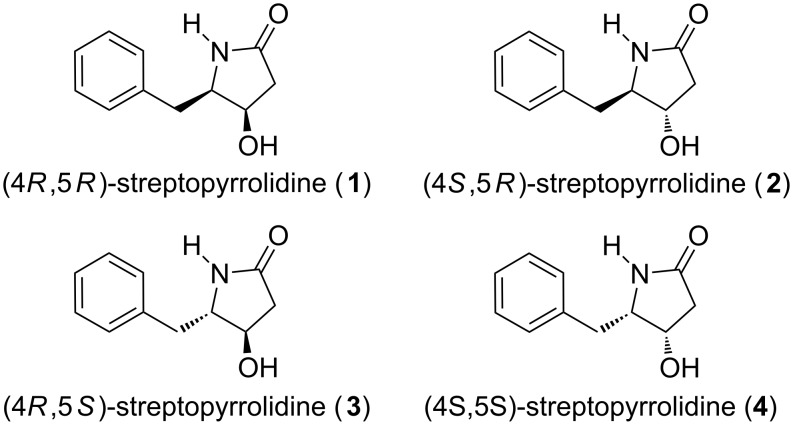
Structures of (4*R,*5*R*)-streptopyrrolidine (**1**), (4*S,*5*R*)-streptopyrrolidine (**2**) (4*R,*5*S*)-streptopyrrolidine (**3**) and (4*S,*5*S*)-streptopyrrolidine (**4**).

Recently, streptopyrrolidine (Figure 1), was isolated as an angiogenesis inhibitor from the fermentation broth of a marine *Streptomyces* sp. found in deep sea sediments [12]. The system is present in many biologically active compounds [13–21] and it could act as a versatile intermediate for the synthesis of a wide range of γ-amino acids as well as pyrrolidines [22–23]. The interesting chemical structure and potent anti-angiogenic activity at non-toxic threshold doses of streptopyrrolidine attracted our attention for developing a concise and efficient protocol for its synthesis in sizable amounts for further biological studies. To date, three syntheses [15,24–25] of (4*S,*5*S*)-streptopyrrolidine have been described, two of which [15,24] were reported only as a synthetic intermediate prior to its isolation from the natural source.

## Results and Discussion

**Scheme 1:**
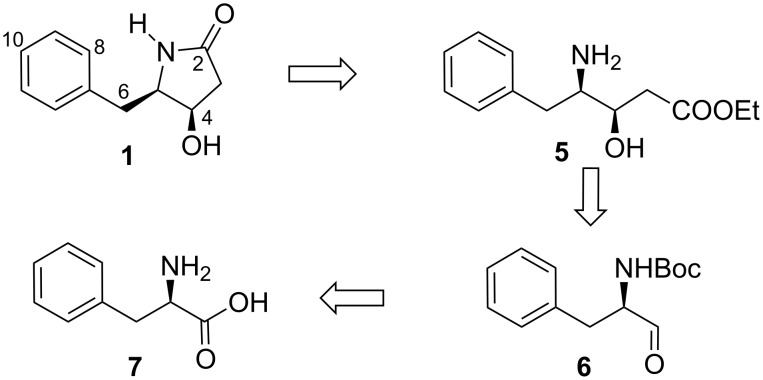
Retrosynthetic analysis.

In this paper, we report the syntheses of (4*R,*5*R*)-streptopyrrolidine (**1**), (4*S,*5*R*)-streptopyrrolidine (**2**), (4*R,*5*S*)-streptopyrrolidine (**3**) and (4*S,*5*S*)-streptopyrrolidine (**4**) in a concise and highly efficient manner via a highly stereoselective aldol type reaction and Lewis acid mediated lactamization as the key reactions. A retrosynthetic analysis for streptopyrrolidine is depicted in Scheme 1.

**Scheme 2:**
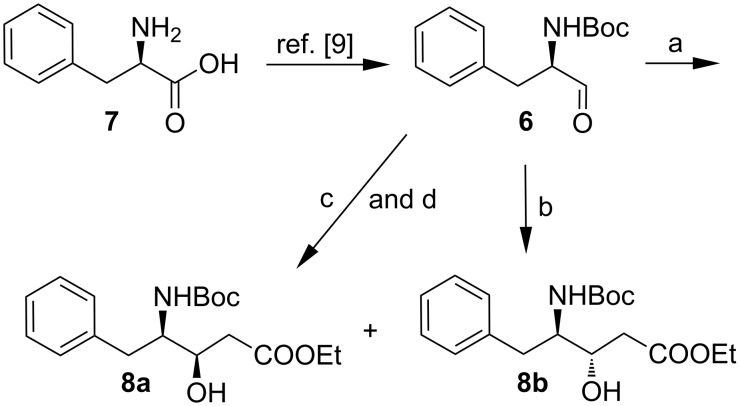
Reagents and conditions: (a) LDA, EtOAc, THF, −78 °C, 4 h, 80% (**8a**:**8b** = 3:2); (b) BF_3_·OEt_2_, (1-ethoxyvinyloxy)trimethylsilane, CH_2_Cl_2_, −78 °C, 2 h, 85% (exclusively **8b**); (c) SnCl_4_, (1-ethoxyvinyloxy)trimethylsilane, CH_2_Cl_2_, −78 °C, 2 h, 70% (**8a**:**8b** = 3:7); (d) LDA, EtOAc, ZnBr_2_, −78 °C, 1 h, 82%, (**8a**:**8b** = 95:5).

The synthesis was initiated from D-phenylalanine (**7**) which was converted to *N*-Boc-D-phenylalaninal (**6**) in 76% yield in three steps by a known protocol [26–27]. The aldehyde was treated with the lithium enolate of ethyl acetate [28–29] at −78 °C according to a modification of the procedure of Steulmann and Klostermeyer [30] to afford two diastereomers **8a** and **8b** in a 3:2 ratio (as determined by NMR) (Scheme 2).

Diastereomers **8a** and **8b** were easily separated by standard column chromatography on silica gel. The pioneering work on catalytic aldol reactions recently reported by Shibasaki [31] prompted us to investigate whether the ketene silyl acetal could be utilized for the aldol reaction in the presence of different Lewis acids to obtain a better selectivity particularly for (*R*)-*tert*-butyl (1-oxo-3-phenylpropan-2-yl)carbamate (**6**). To our surprise, when the reaction was carried out with the ketene silyl acetate at −78 °C, of the five Lewis acids investigated (SnCl_4_, BF_3_·OEt_2_, ZnI_2_, TiCl_4_, EtOAc/LDA/ZnBr_2_) BF_3_·OEt_2_ gave excellent selectivity (exclusively **8b**) (Table 1).

**Table 1:** Addition reaction under different conditions to obtain **8a** and **8b.**

Entry	Reagent	Additive (Lewis Acid)	Time (h)	Yield^a^ (%)	**8a**:**8b**

1	ketene silyl acetate	SnCl_4_	2	70	30:70
2	ketene silyl acetate	BF_3_·OEt_2_	2	85	0:100
3	ketene silyl acetate	TiCl_4_	10	42	35:65
4	ketene silyl acetate	ZnI_2_	12	59	80:20
5	EtOAC/LDA	ZnBr_2_	1	82	95:05

^a^isolated yield.

**Figure 2:**
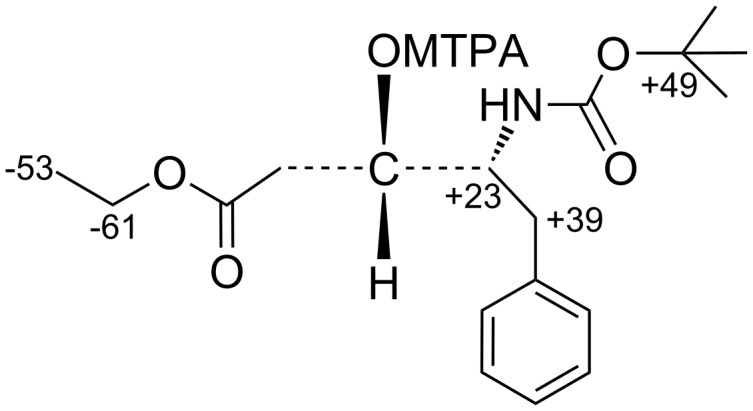
Δδ = (δ_S_−δ_R_) × 10^3^ for (*S*)- and (*R*)-MTPA esters of compound **8b**.

Following a modification of the Mosher method [32–33], the newly created stereogenic center in compound **8b** bearing the hydroxyl group was assigned. The syntheses of both the (*S*)- and (*R*)-MTPA ester of **8b** were achieved using MTPA acid with DCC as the coupling reagent. The chemical shifts of both the (*S*)- and (*R*)-MTPA esters of **8b** were assigned by ^1^H NMR. From the equation given in Figure 2, the Δδ values were calculated for as many protons as possible. The carbon chain bearing protons showing Δδ negative values should be placed on the left hand side of the model (Figure 2) whilst that where Δδ has positive values should be placed on the right hand side. From this the center was found to have the *S*-configuration which thus establishes the absolute stereochemistry of **8a**. With the absolute stereochemistry of both the isomers known, we were interested to develop a protocol to have control over the selectivity to obtain exclusively **8a**. Stereoselective addition of the zinc enolate [34] of ethyl acetate to *N*-Boc-phenylalaninal (**6**) resulted in **8a** as a 95:5 mixture of diastereomers (as determined by HPLC), which were easily separated by silica gel column chromatography. The stereochemical outcome in both the cases was explained by Crams’ rule, i.e., for a non-chelation model, the (*R*)-aldehyde would give rise to the (3*S*,4*R*) isomer. Alternatively, in a chelation model, the metal imposes a *syn* relationship between the formyl and neighboring nitrogen functionality which would lead to the (3*R*,4*R*) isomer [35].

**Scheme 3:**
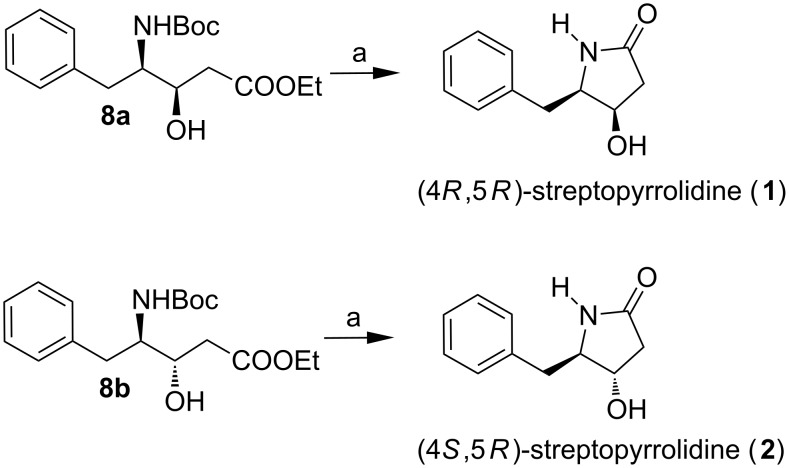
Reagents and conditions: (a) (1) TFA, CH_2_Cl_2_, rt, 4 h; (2) Zr(O*^t^*Bu)_4_, HOAt, toluene, 60 °C, 12 h, 82% over two steps.

Deprotection of the Boc-group with TFA and CH_2_Cl_2_ was followed by evaporation and treatment of the crude product under different reaction conditions for lactamization (Table 2 is for lactamization of **8a**, almost similar yields were obtained for **8b**), afforded (4*R,*5*R*)-streptopyrrolidine (**1**) and (4*S,*5*R*)-streptopyrrolidine (**2**) in good to excellent overall yield (Scheme 3). From all of the conditions investigated, Zr(O*^t^*Bu)_4_ in presence of 1-hydroxy-7-azabenzotriazole (HOAt) gave the best result [36], i.e., 82% yield over the two steps. The spectral and analytical data {[α]_D_^25^ +40.5 (*c* 1.8, MeOH); lit. [15,25] [α]_D_^25^ −43.5 (*c* 1.0, MeOH) for its enantiomer} of compound **1** [6] were in good agreement with the reported values for its enantiomer except the sign of the specific rotation, which confirmed the absolute configuration of the natural product as (4*S*,5*S*). To confirm further the absolute stereochemistry of natural streptopyrrolidine, (4*R,*5*S*)-streptopyrrolidine (**3**) and (4*S,*5*S*)-streptopyrrolidine (**4**) were prepared starting from L-phenylalanine. The spectral and analytical data of (4*S,*5*S*)-streptopyrrolidine were in good agreement with natural streptopyrrolidine.

**Table 2:** Lactamization under different reaction conditions to obtain **1** and **2.**

Entry	Reagent	Additive/ Solvent	Temp. (°C)	Time (h)	Yield^a^ (%)

1	Py	none/CH_2_Cl_2_	50	12	41
2	Et_3_N	none/CH_2_Cl_2_	50	12	46
3	Py	none/toluene	100	6	55
4	Et_3_N	none/toluene	100	6	58
5	KO*^t^*Bu	HOAt/toluene	60	6	42
6	Zr(O*^t^*Bu)_4_	HOAt/toluene	60	6	82

^a^yield over two steps.

## Conclusion

In conclusion, the total synthesis of all four possible isomers of streptopyrrolidine (**1**–**4**) has been achieved in ≈42% yield over 6 steps starting from either D- or L-phenylalanine which further confirmed the absolute configuration of natural streptopyrrolidine. Our protocol is highly flexible for the synthesis of all four possible isomers of streptopyrrolidine compared to previous reports.

## Experimental

### 

#### General information

All reactions were carried out under an inert atmosphere, unless otherwise stated. Solvents were dried and purified by standard methods prior to use. The progress of all reactions was monitored by TLC using glass plates pre-coated with silica gel 60 F254 with a thickness of 0.5 mm. Column chromatography was performed on silica gel (60 mesh) with ethyl acetate and hexane the eluent. Optical rotations were measured with a Perkin Elmer P241 polarimeter and a JASCO DIP-360 digital polarimeter at 25 °C. IR spectra were recorded on a Perkin-Elmer FT-IR spectrometer. ^1^H and ^13^C NMR spectra were recorded on a Variant Gemini 200 MHz, Bruker Avance 300 MHz, or Varian Inova 500 MHz spectrometer with TMS as an internal standard in CDCl_3_, CD_3_OD etc. Mass spectra were recorded on a Micromass VG-7070H for EI.

**(3*R*,4*R*)-Ethyl-4-(*tert*-butoxycarbonylamino)-3-hydroxy-5-phenylpentanoate (8a) and (3*S*,4*R*)-ethyl-4-(*tert*-butoxycarbonylamino)-3-hydroxy-5-phenylpentanoate (8b):** To a stirred solution of the aldehyde **6** (0.2 g, 0.8 mmol) in CH_2_Cl_2_ (5 mL) under a nitrogen atmosphere and cooled to −78 °C, was added 1 M solution of SnCl_4_ in CH_2_Cl_2_ (0.1 mL, 0.81 mmol). After 10 min, (1-ethoxyvinyloxy)trimethylsilane (0.26 g, 1.6 mmol) was added at the same temperature. The reaction mixture was stirred for 1 h at −78 °C. After completion of the reaction (as determined by TLC), the reaction mixture was quenched with 1 N KOH (3 mL). The reaction mixture was extracted with ethyl acetate (3 × 25 mL). The combined organic layers were washed with brine, dried over Na_2_SO_4_ and concentrated under reduced pressure to obtain pale yellow liquid which on purification by silica gel column chromatography afforded **8a** and **8b** in a ratio of 3:7 (0.19 g, 70%) as colorless viscous liquid.

To a stirred solution of the aldehyde **6** (0.1 g, 0.4 mmol) in CH_2_Cl_2_ (5 mL) under a nitrogen atmosphere and cooled to −78 °C, was added BF_3_·OEt_2_ (0.03 g, 0.2 mmol). After 30 min, (1-ethoxy vinyloxy)trimethylsilane (0.26 g, 1.6 mmol) was added at the same temperature. The reaction mixture was stirred for 1 h at −78 °C when TLC showed completion of the reaction. The reaction mixture was quenched with saturated aqueous Na_2_CO_3_, extracted with ethyl acetate (3 × 25 mL). The combined organic layers were washed with brine, dried over Na_2_SO_4_ and concentrated under reduced pressure to give a pale yellow liquid. The crude product was purified by silica gel column chromatography with ethyl acetate and hexane (1:3) as eluent to yield **8b** as a colorless viscous liquid as the sole product (0.115 g, 85%).

A stirred solution of LDA (4.9 mL, 2 N, 9.8 mmol) in anhydrous THF (10 mL) was cooled to −78 °C under nitrogen atmosphere. Ethyl acetate (1.05 mL, 9.8 mmol) was then added followed by a 0 °C solution of anhydrous ZnBr_2_ (2.17 g, 9.8 mmol) in anhydrous THF (5 mL). A −78 °C solution of *N*-Boc-protected aldehyde **6** (0.35 g, 1.41 mmol) in anhydrous THF (4 mL) was added and the mixture stirred at −78 °C for 30 min then allowed to warm to room temperature. The reaction mixture was stirred at room temperature for 10 h. After completion of the reaction (as determined by TLC), saturated NH_4_Cl/acetic acid (9:1) (20 mL) was added to the reaction mixture which was then extracted with ethyl acetate (3 × 30 mL). The combined organic layers were washed with brine (2 × 50 mL), dried over Na_2_SO_4_, concentrated under reduced pressure, and the crude product purified by silica gel column chromatography to give **8a** (0.378 g, 82%) as a colorless viscous liquid. **Analytical and spectral data of 8a:** [α]_D_^25^ +34.9 (*c* 1.2, MeOH); IR (KBr): 3553, 3496, 3376, 2979, 2934, 1727, 1683 cm^−1^; ^1^H NMR (300 MHz, CDCl_3_): 7.28–7.20 (m, 5H, Ar*H*), 5.07 (d, *J* = 9.6 Hz, 1H, N*H*), 4.16–4.09 (q, *J* = 6.9, 14.1 Hz, 2H, OC*H_2_*CH_3_), 3.98 (d, *J* = 9.6 Hz, 1H, C*H*OH), 3.74 (q, *J* = 8.1, 16.2 Hz, 1H, C*H*NH), 2.91 (d, *J* = 7.5 Hz, 2H, PhC*H_2_*), 2.59 (dd, *J* = 10.1, 16.8 Hz, 1H, C*H'*_2_COO), 2.38 (dd, *J* = 2.3, 16.9 Hz, 1H, C*H*_2_COO), 1.41 (s, 9H, *t*-Bu), 1.23 (t, *J* = 6.9 Hz, 3H, OCH_2_C*H_3_*); ^13^C NMR (75 MHz, CDCl_3_): 173.4, 155.8, 138.1, 129.3, 128.4, 126.3, 79.3, 67.0, 60.7, 55.3, 38.5, 29.6, 28.3, 14.0; ESI-MS: *m*/*z* = 338 [M + H]^+^; ESI-HRMS: Calcd. for C_18_H_27_NNaO_5_, 360.1781; found: 360.1789. **Analytical and spectral data of 8b:** [α]_D_^25^ +11.6 (*c* 0.6, MeOH); IR (KBr): 3354, 2982, 2936, 1735, 1684 cm^−1^; ^1^H NMR (300 MHz, CDCl_3_) 7.31–7.20 (m, 5H, Ar*H*), 4.61 (d, *J* = 8.4 Hz, 1H, NH), 4.20–4.13 (q, *J* = 7.1, 14.1 Hz, 2H, OC*H_2_*CH_3_), 3.99–3.86 (m, 2H, C*H*OH, C*H*NH), 2.96 (dd, *J* = 3.8, 13.7 Hz, 1H, PhC*H'*_2_), 2.82 (m, 1H, PhC*H*_2_), 2.64–2.45 (m, 2H, C*H*_2_COO), 1.34 (s, 9H, *t*-Bu), 1.25 (t, *J* = 7.1 Hz, 3H, OCH_2_C*H_3_*); ^13^C NMR (75 MHz, CDCl_3_): 172.9, 155.7, 137.6, 129.4, 128.4, 126.4, 79.6, 70.1, 60.8, 55.1, 38.2, 35.8, 28.2, 14.1; ESI-MS: *m*/*z* = 360 [M + Na]^+^; ESI-HRMS: Calcd. for C_18_H_27_NNaO_5_, 360.1781; found: 360.1794.

**(4*R*,5*R*)-streptopyrrolidine (1), (4*S*,5*R*)-streptopyrrolidine (2), (4*R*,5*S*)-streptopyrrolidine (3), (4*S*,5*S*)-streptopyrrolidine (4):** A solution of TFA/H_2_O (2.8 mL, 0.28 mL) was added to **8a** or **8b** (200 mg, 0.59 mmol) and the resulting mixture stirred at room temperature for 3 h. After completion of the reaction (as determined by TLC), the solvent was evaporated under reduced pressure and the resulting reddish oil dissolved in ethyl acetate (10 mL). The organic phase was washed with NaHCO_3_, dried over Na_2_SO_4_ and concentrated under reduced pressure. The red oil so obtained was dissolved in toluene. Zr(O*^t^*Bu)_4_ (22 mg, 0.059 mmol) followed by HOAt (160 mg, 0.11 mmol) were added and the reaction mixture allowed to stir at 60 °C for 12 h. After completion of the reaction (as determined by TLC), the toluene was evaporated under reduced pressure*.* Water was added and the reaction mixture extracted with CH_2_Cl_2_ (3 × 20 mL). The combined organic layers were dried over Na_2_SO_4_ and concentrated under reduced pressure to give a brown liquid which on purification by silica gel column chromatography furnished **1** (98 mg, 82%) as a white sticky solid. **Analytical and spectral data of 1:** [α]_D_^25^ +40.5 (*c* 1.8, MeOH); IR (KBr): 3452, 3234, 2924, 1690 cm^−1^; ^1^H NMR (500 MHz, DMSO-*d*_6_): 7.51 (s, 1H, NH), 7.27 (m, 4H, ArH), 7.17 (m, 1H, ArH), 5.14 (d, *J* = 4.1 Hz, 1H, OH), 4.10 (s, 1H, H-4), 3.67 (q, *J* = 5.6 Hz, 1H, H-5), 2.96 (dd, *J* = 7.9, 13.4 Hz, 1H, NCHC*H'*_2_), 2.65 (dd, *J* = 6.2, 13.4 Hz, 1H, NCHC*H*_2_), 2.38 (dd, *J* = 6.1, 16.4 Hz, 1H, H-3'), 1.96 (dd, *J* = 2.6, 16.4 Hz, 1H, H-3); ^13^C NMR (75 MHz, DMSO-*d*_6_): 174.8, 138.6, 129.2, 128.1, 125.9, 66.9, 60.0, 40.8, 34.5; EIMS: 192 [M+H]^+^; ESI-HRMS: Calcd. for C_11_H_14_NO_2_, 192.1019; found: 192.1026. **Analytical and spectral data of 2:** [α]_D_^25^ −13.2 (*c* 1.6, MeOH); IR (neat): 3384, 2925, 2855, 1679 cm^−1^; ^1^H NMR (500 MHz, DMSO-*d*_6_): 7.68 (s, 1H, NH), 7.29–7.21 (m, 5H, ArH), 5.13 (d, *J* = 3.9 Hz, 1H, OH), 3.95 (s, 1H, H-4), 3.51 (t, *J* = 6.8 Hz, 1H, H-5), 2.69 (m, 2H, NCHC*H*_2_), 2.27 (dd, *J* = 6.8, 17.5 Hz, 1H, H-3'), 1.80 (dd*, J* = 1.9, 16.5 Hz, 1H, H-3); ^13^C NMR (75 MHz, DMSO-*d*_6_): 174.8, 137.6, 129.4, 128.4, 126.2, 69.3, 63.4, 40.0, 38.9; ESI-HRMS: Calcd. for C_11_H_14_NO_2_, 192.1019; found: 192.1024. **Analytical and spectral data of 3:** [α]_D_^25^ +12.6 (*c* 1.2, MeOH); IR (KBr): 3462, 2928, 1784, 1730,1450, 1147, 1069, 744.8 cm^−1^; ^1^H NMR (300 MHz, DMSO-d_6_): δ 7.70 (s, 1H, NH), 7.18–7.32 (m, 5H, ArH), 5.14 (d, *J* = 3.7 Hz, 1H, OH), 3.95 (s, 1H, H-4)*,* 3.51 (q, *J* = 6.0, 13.2 Hz, 1H, H-5), 2.67 (d, *J* = 2.2 Hz, 2H, NCHC*H*_2_), 2.27 (dd, 1H, *J* = 6.0, 16.6 Hz, 1H, H-3'), 1.82 (dd, *J* = 2.2, 16.6 Hz, 1H, H-3); ^13^C NMR (75 MHz, DMSO-*d*_6_): 174.8, 137.6, 129.4, 128.2, 126.0, 69.2, 63.4, 39.9, 39.1; ESI-HRMS: Calcd. for C_11_H_14_NO_2_, 192.1019; found: 192.1026. **Analytical and spectral data of 4:** [α]_D_^25^ −41.2 (*c* 1.0, MeOH); IR (KBr): 3451, 1785, 1453, 1185, 1147, 1058, 786, 698 cm^−1^; ^1^H NMR (300 MHz, DMSO-*d*_6_): δ 7.55 (s, 1H, NH), 7.08–7.37 (m, 5H, ArH), 5.18 (d, *J* = 3.8 Hz, 1H, OH), 4.09(s, 1H, H-4), 3.67 (s, 1H, H-5), 2.96 (dd, *J* = 7.1, 12.6 Hz, 1H, NCHC*H*_2_'), 2.66 (dd, *J* = 8.0, 16.9 Hz, 1H, NCHC*H*_2_), 2.38 (dd, *J* = 6.1, 15.6 Hz, 1H, H-3'), 1.99 (dd, *J* = 3.0, 15.6 Hz, 1H, H-3); ^13^C NMR (75 MHz, DMSO-*d*_6_): 174.9, 138.6, 129.2, 128.2, 126.8, 66.9, 60.1, 40.8, 34.5; ESI-HRMS: Calcd. for C_11_H_14_NO_2_, 192.1019; found: 192.1026.

## Supporting Information

Supporting Information features ^1^H and ^13^C NMR spectra of all intermediates.

File 1 ^1^H and ^13^C NMR spectra of all intermediates.
