# Supplementary material for: Stereoselective synthesis of four possible isomers of streptopyrrolidine
Source: Beilstein J Org Chem. 2011 Jan 10;7:34–9. doi: 10.3762/bjoc.7.6 (PMC3028605; doi:10.3762/bjoc.7.6)
Supplement: File 1 — 1H and 13C NMR spectra of all intermediates. [file Beilstein_J_Org_Chem-07-34-s001.pdf]

Supporting Information

for

Stereoselective synthesis of four possible isomers of streptopyrrolidine

Debendra K. Mohapatra*, Barla Thirupathi, Pragna P. Das and Jhillu S. Yadav*

Address: Division of Organic Chemistry-I, Indian Institute of Chemical Technology (CSIR), Hyderabad-500607, India, Tel/Fax: 0091-40-27193128

Email: Debendra K. Mohapatra - mohapatra@iict.res.in

Jhillu S. Yadav - yadavpub@iict.res.in

^{1}H and ^{13}C NMR spectra of all intermediates

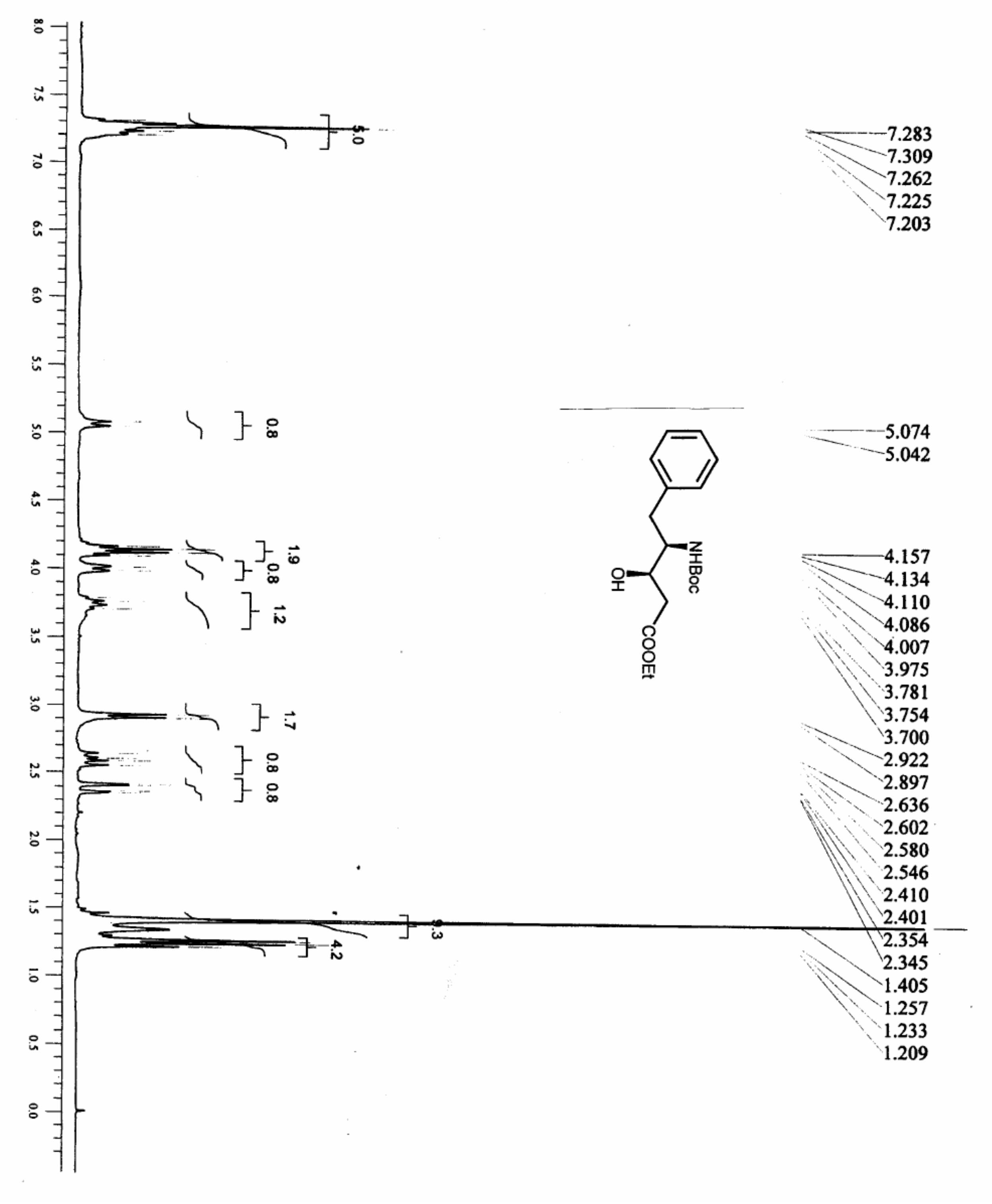
7.283
7.309
7.262
7.225
7.203
5.074
5.042
4.157
4.134
4.110
4.086
4.007
3.975
3.781
3.754
3.700
2.922
2.897
2.636
2.602
2.580
2.546
2.410
2.401
2.354
2.345
1.405
1.257
1.233
1.209
NHBoc
OH
COOEt

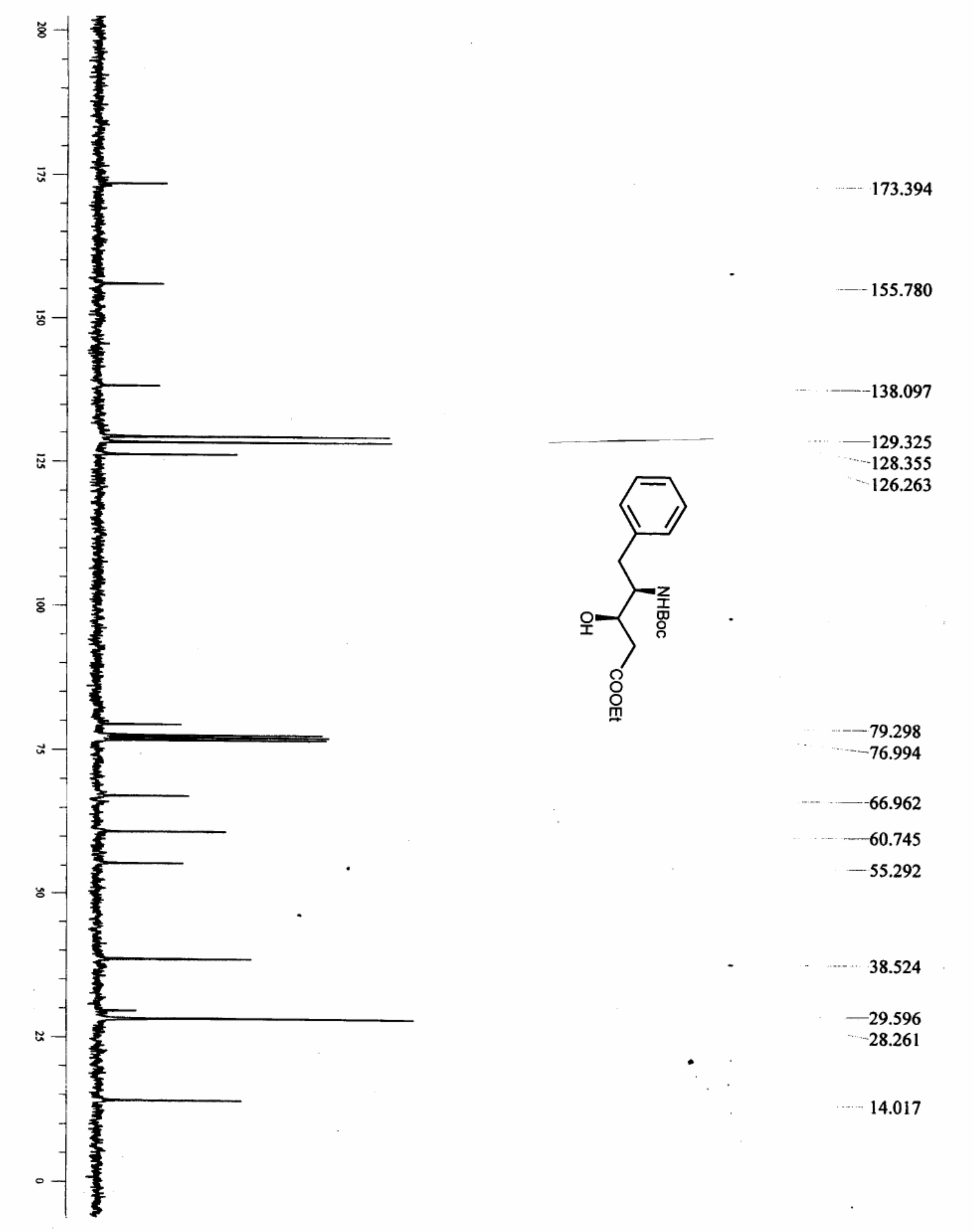
173.394
155.780
138.097
129.325
128.355
126.263
NHBoc
OH
COOEt
79.298
76.994
66.962
60.745
55.292
38.524
29.596
28.261
14.017

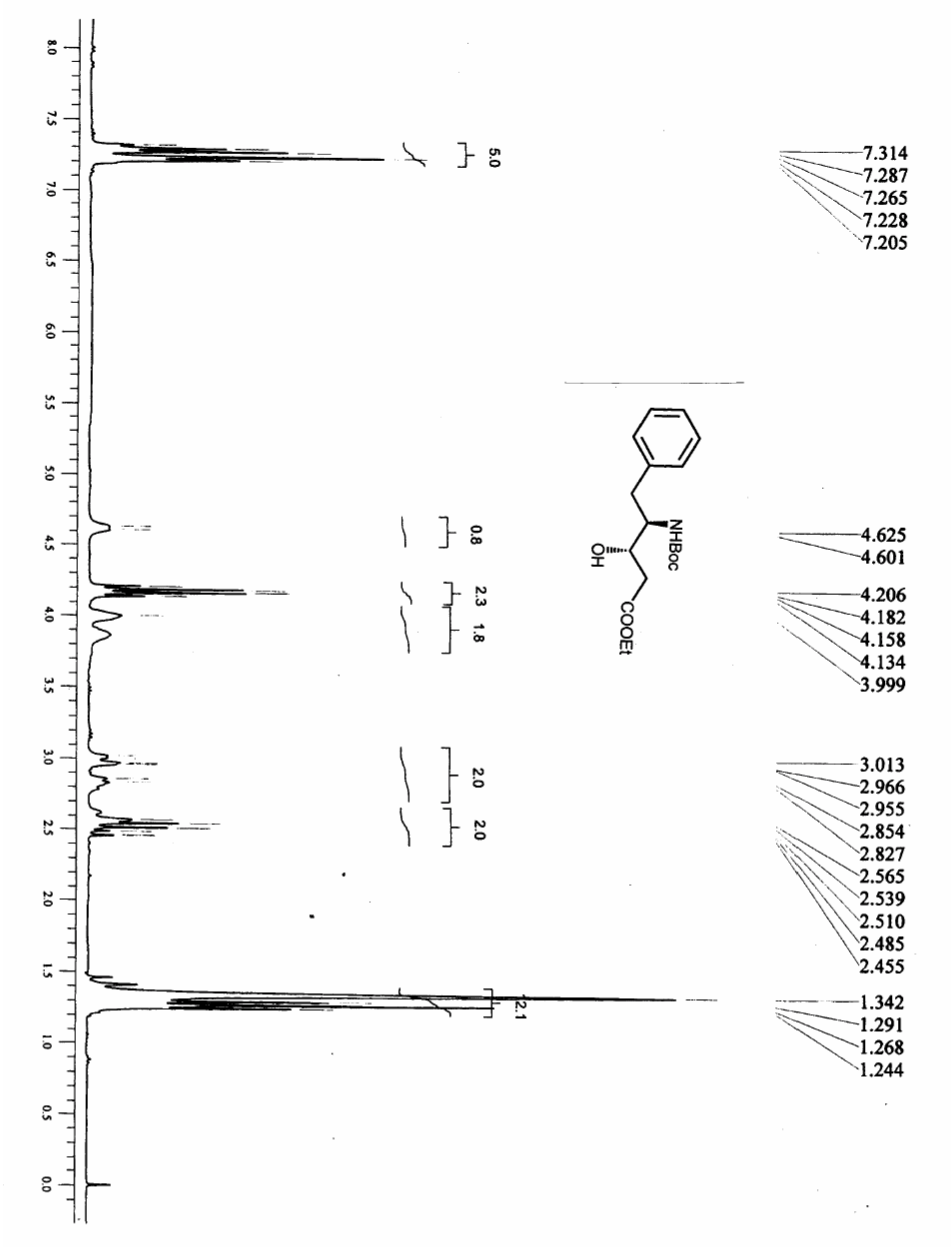
NHBoc
OH
COOEt
7.314
7.287
7.265
7.228
7.205
4.625
4.601
4.206
4.182
4.158
4.134
3.999
3.013
2.966
2.955
2.854
2.827
2.565
2.539
2.510
2.485
2.455
1.342
1.291
1.268
1.244
5.0
0.8
2.3
1.8
2.0
2.0
2.1
8.0
7.5
7.0
6.5
6.0
5.5
5.0
4.5
4.0
3.5
3.0
2.5
2.0
1.5
1.0
0.5
0.0

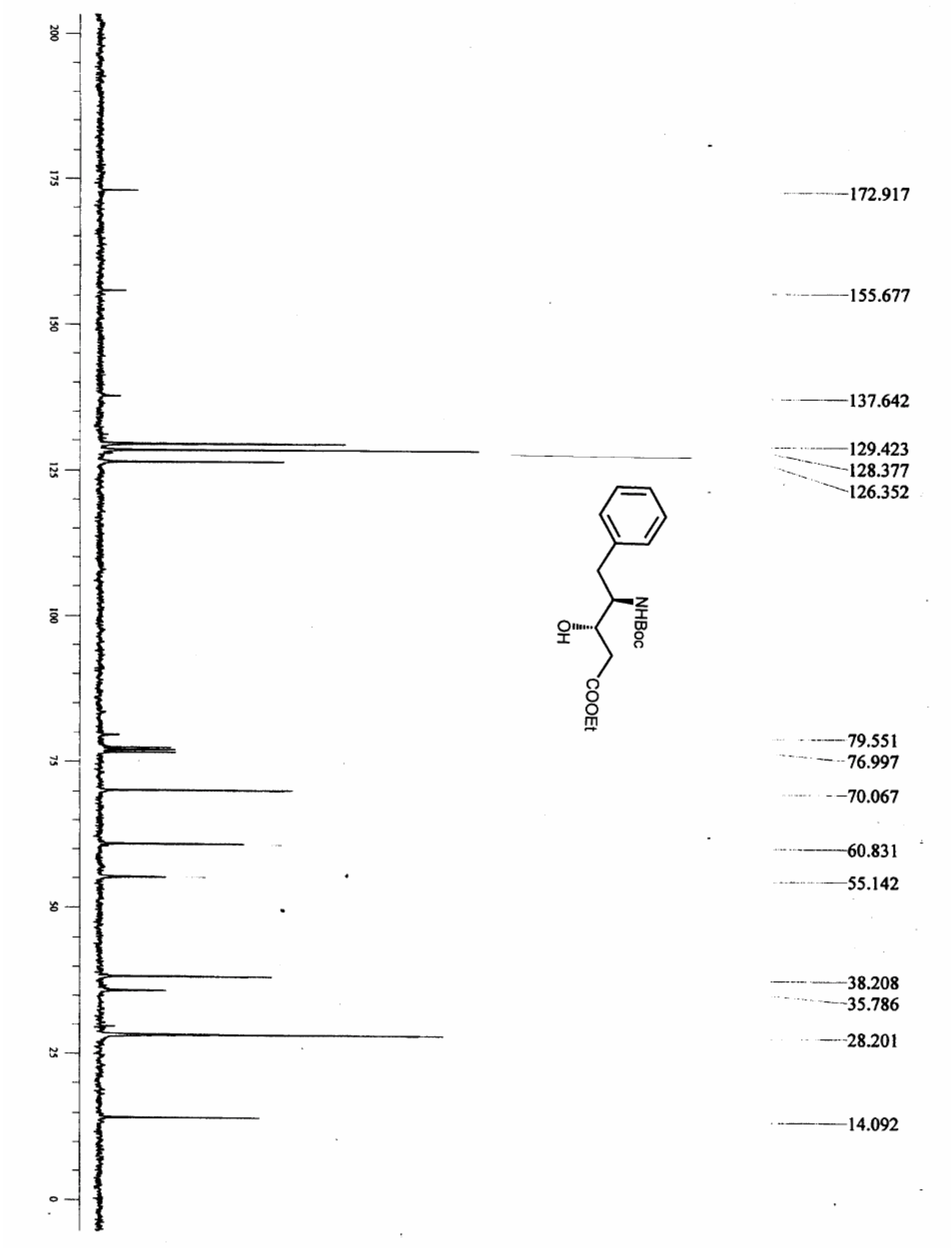

172.917
155.677
137.642
129.423
128.377
126.352
NHBoc
OH
COOEt
79.551
76.997
70.067
60.831
55.142
38.208
35.786
28.201
14.092

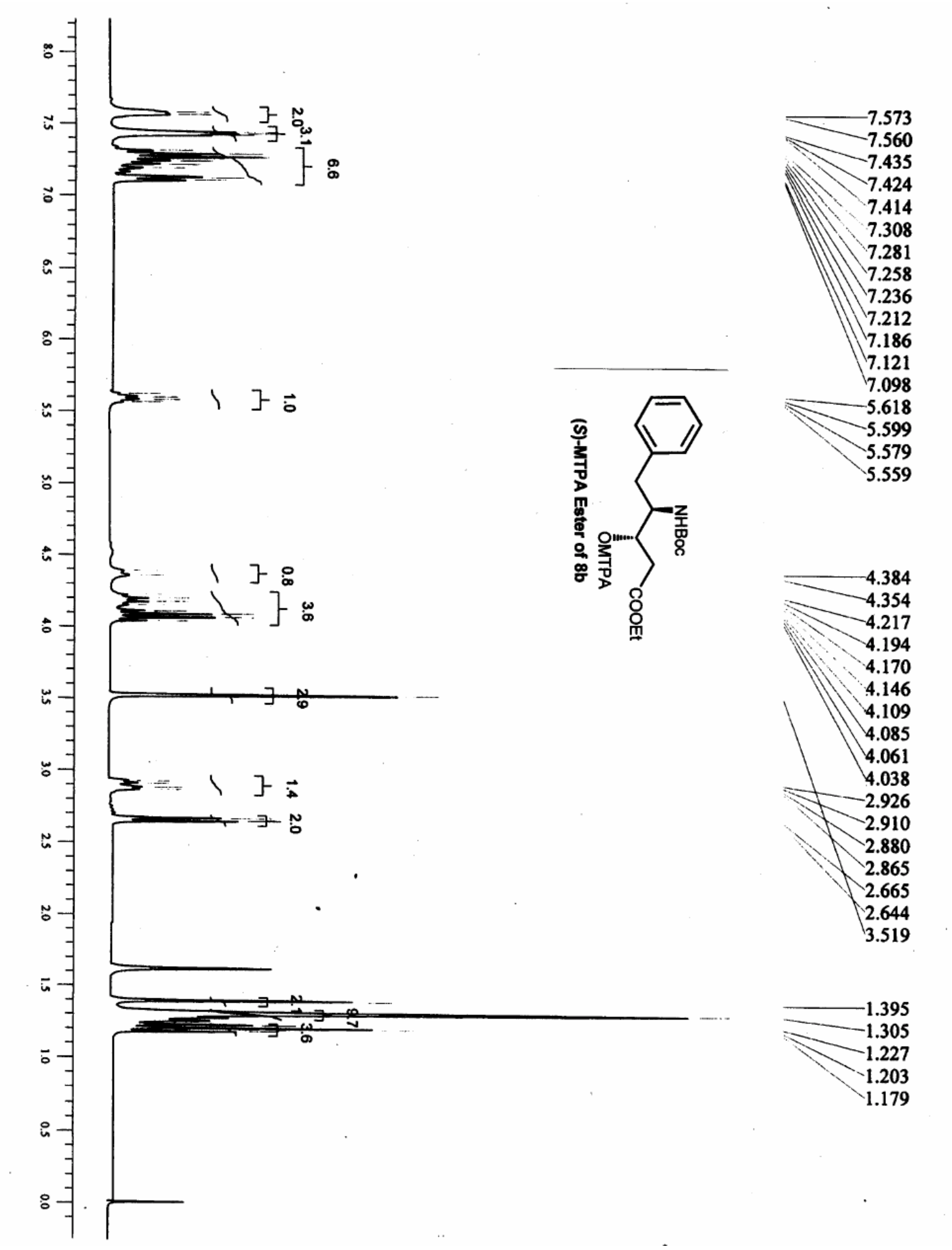

(S)-MTPA Ester of 8b
NHBoc
OMTPA
COOEt
7.573
7.560
7.435
7.424
7.414
7.308
7.281
7.258
7.236
7.212
7.186
7.121
7.098
5.618
5.599
5.579
5.559
4.384
4.354
4.217
4.194
4.170
4.146
4.109
4.085
4.061
4.038
2.926
2.910
2.880
2.865
2.665
2.644
3.519
1.395
1.305
1.227
1.203
1.179

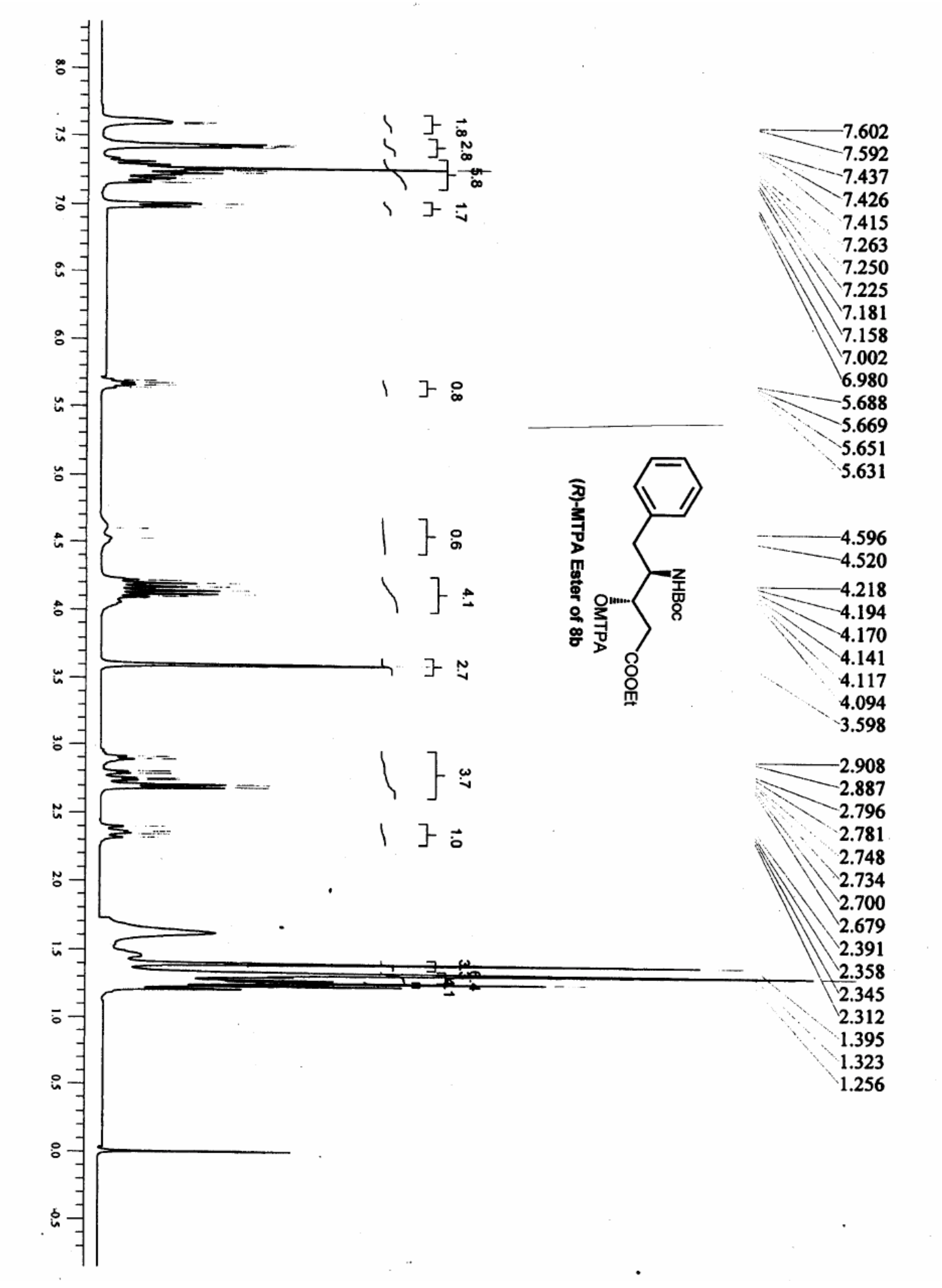

(R)-MTPA Ester of 8b
NHBoc
OMTPA
COOEt
7.602
7.592
7.437
7.426
7.415
7.263
7.250
7.225
7.181
7.158
7.002
6.980
5.688
5.669
5.651
5.631
4.596
4.520
4.218
4.194
4.170
4.141
4.117
4.094
3.598
2.908
2.887
2.796
2.781
2.748
2.734
2.700
2.679
2.391
2.358
2.345
2.312
1.395
1.323
1.256

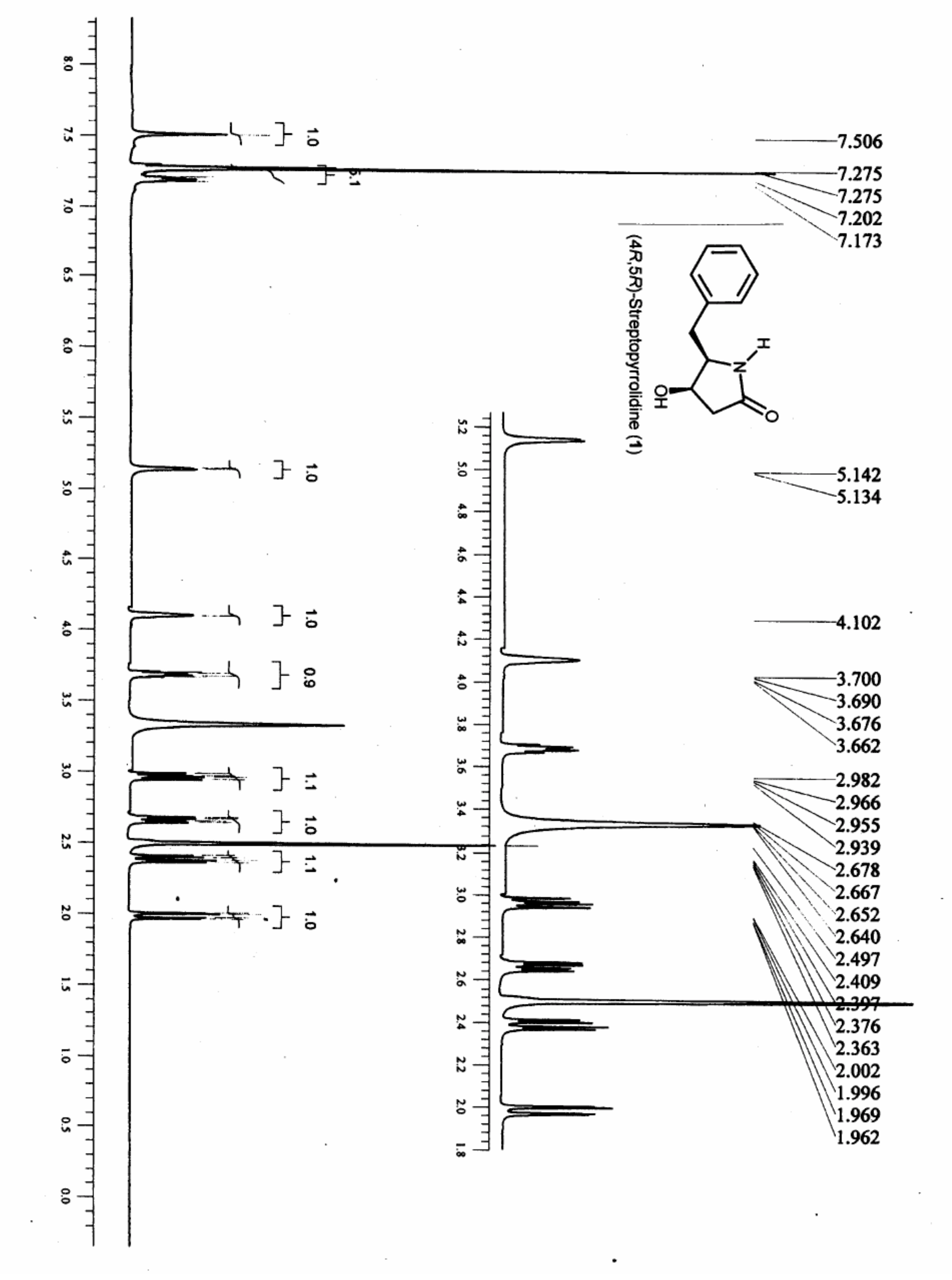
(4R,5R)-Streptopyrrolidine (1)
7.506
7.275
7.275
7.202
7.173
5.142
5.134
4.102
3.700
3.690
3.676
3.662
2.982
2.966
2.955
2.939
2.678
2.667
2.652
2.640
2.497
2.409
2.397
2.376
2.363
2.002
1.996
1.969
1.962

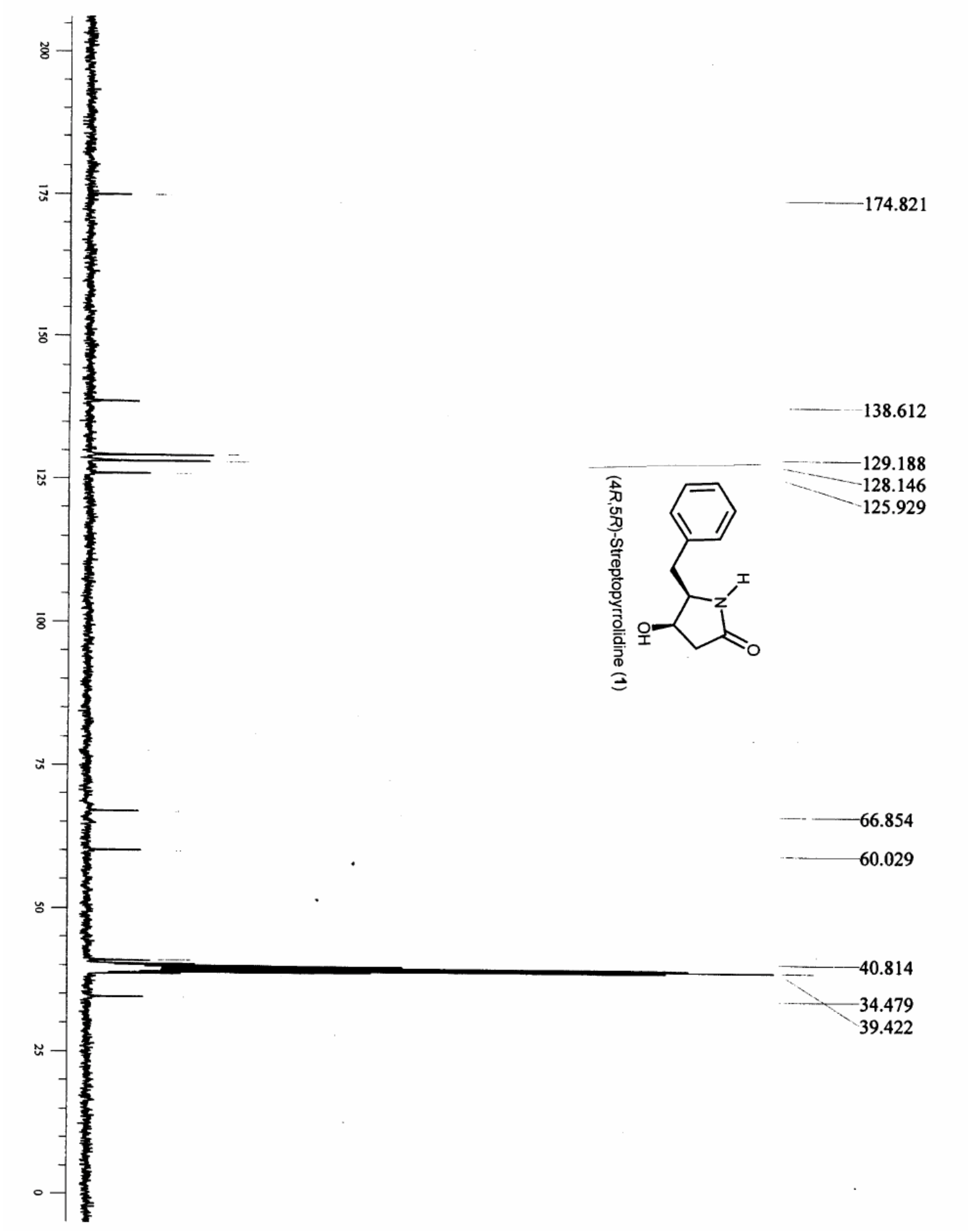
174.821
138.612
129.188
128.146
125.929
66.854
60.029
40.814
34.479
39.422
(4R,5R)-Streptopyrrolidine (1)
200
175
150
125
100
75
50
25
0

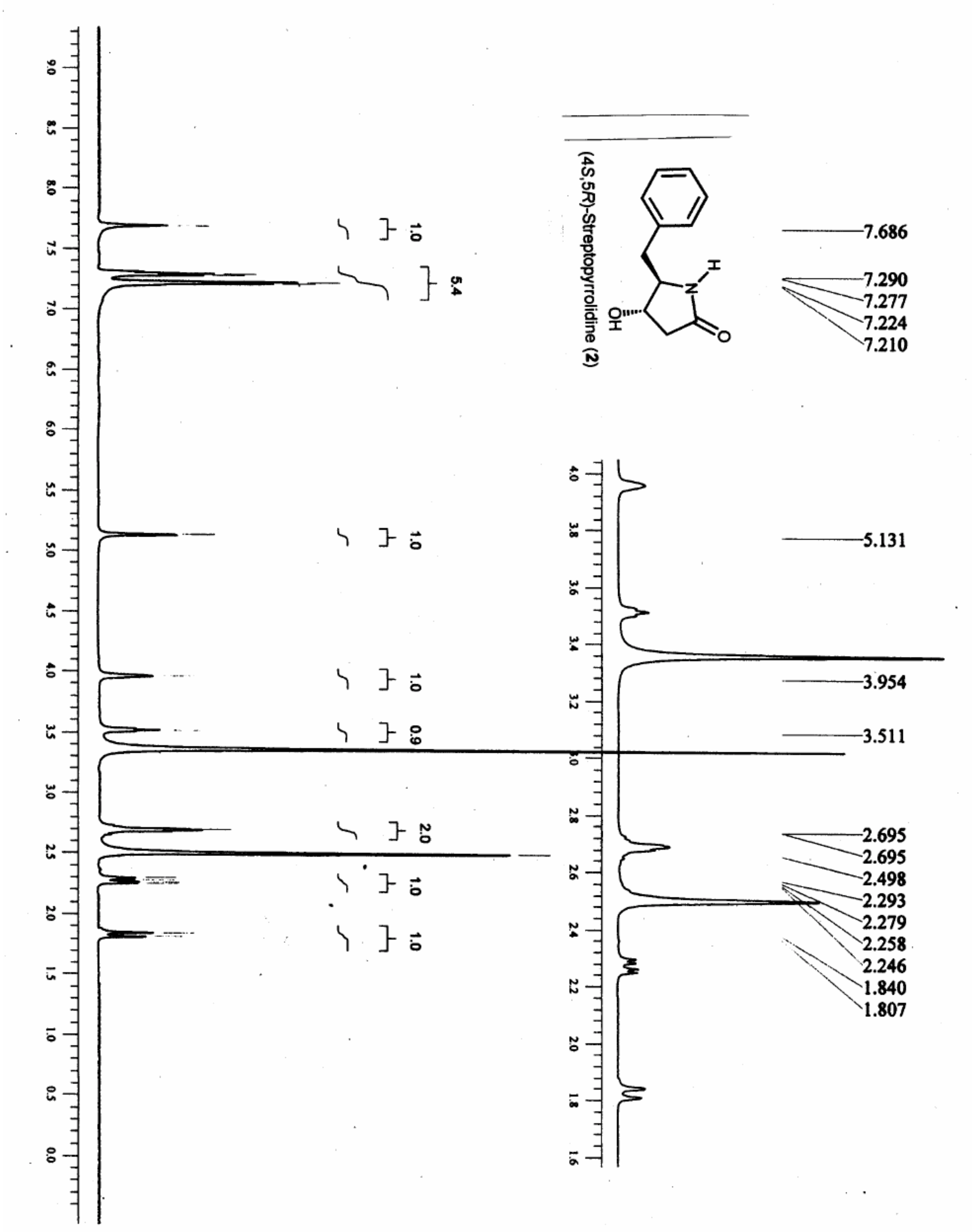
(4S,5R)-Streptopyrrolidine (2)
7.686
7.290
7.277
7.224
7.210
5.131
3.954
3.511
2.695
2.695
2.498
2.293
2.279
2.258
2.246
1.840
1.807
1.0
5.4
1.0
1.0
0.9
2.0
1.0
1.0

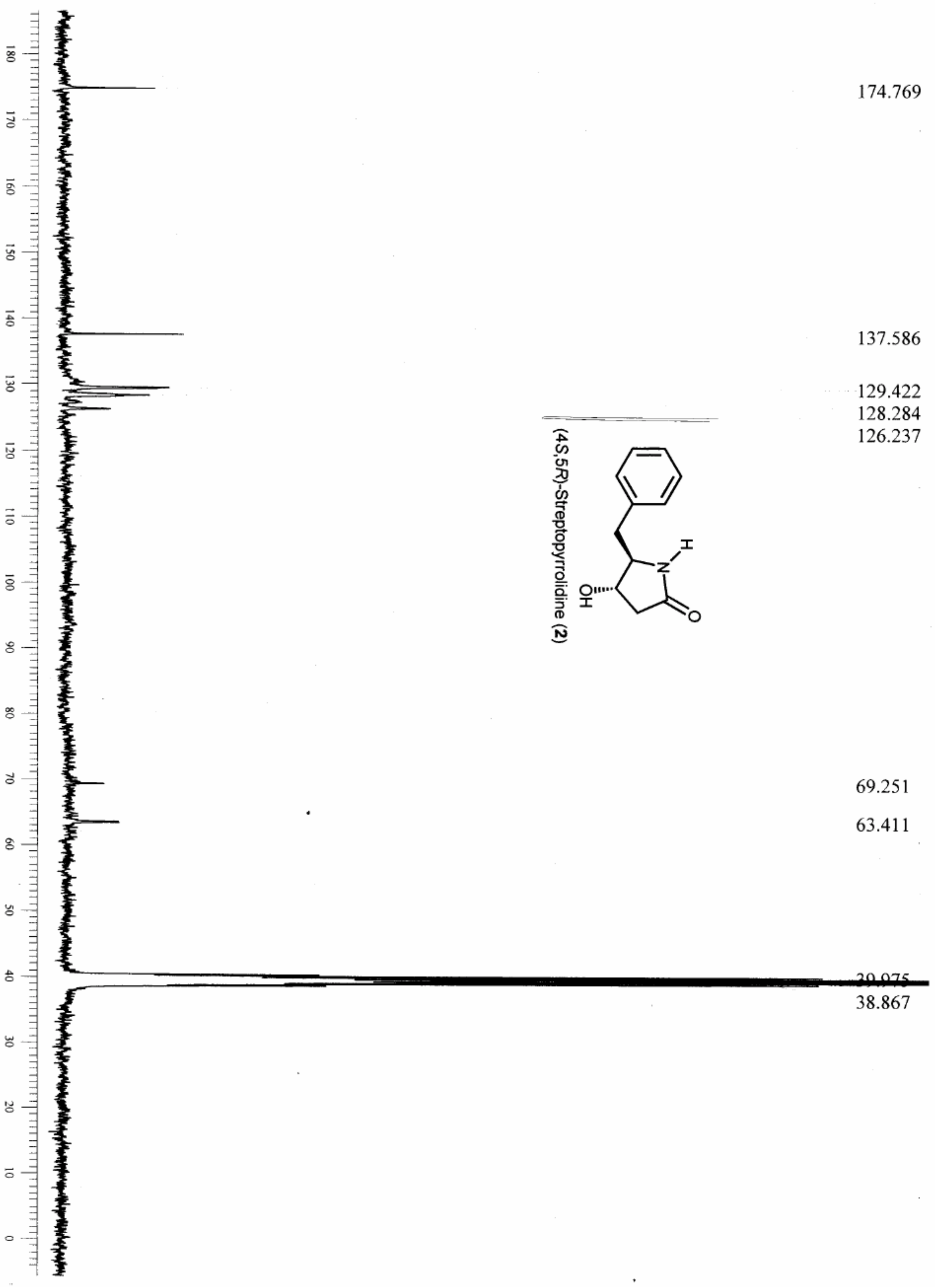
174.769
137.586
129.422
128.284
126.237
69.251
63.411
39.975
38.867
(4S,5R)-Streptopyrrolidine (2)

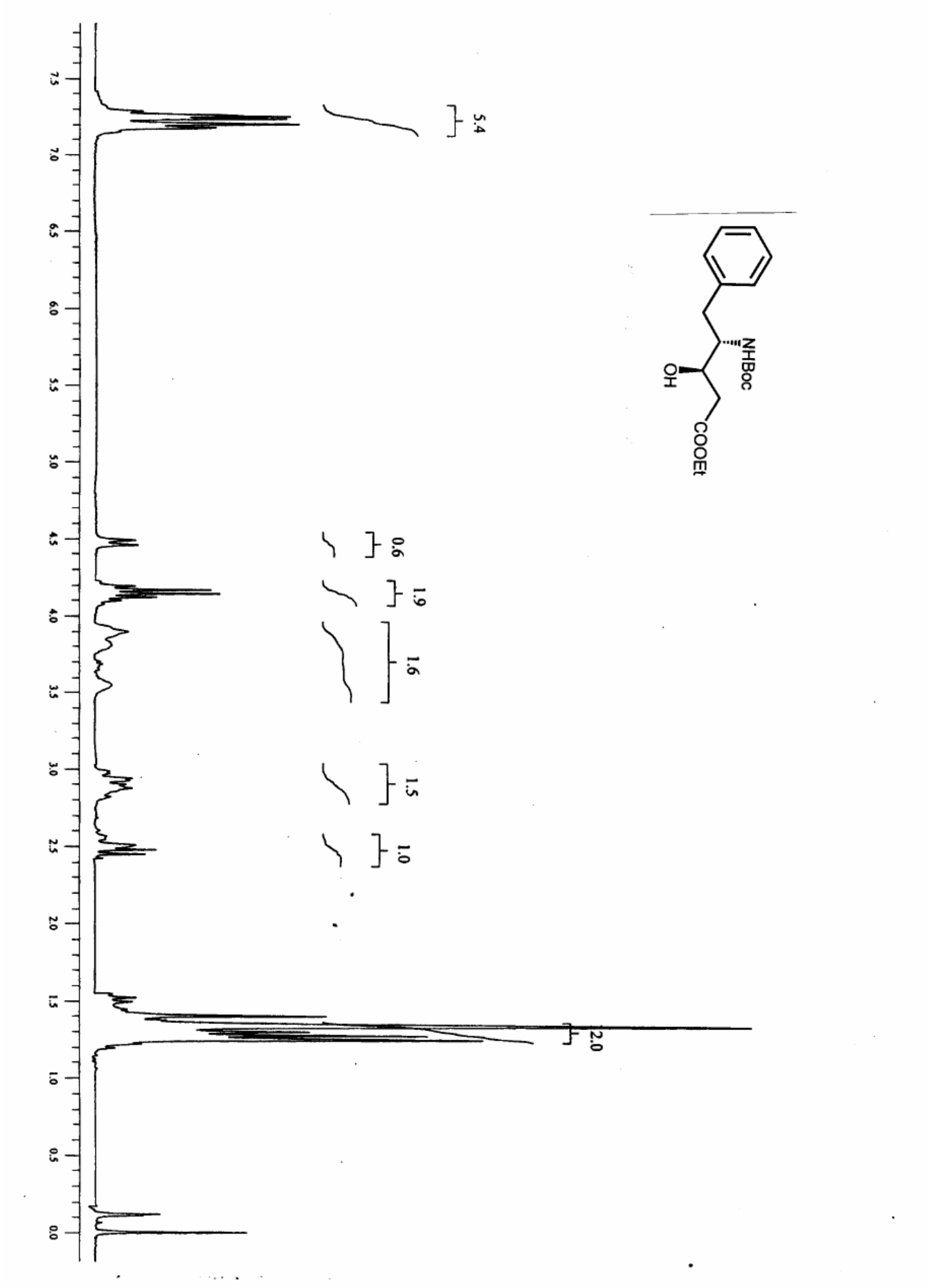

7.5
7.0
6.5
6.0
5.5
5.0
4.5
4.0
3.5
3.0
2.5
2.0
1.5
1.0
0.5
0.0
5.4
0.6
1.9
1.6
1.5
1.0
2.0
NHBoc
OH
COOEt

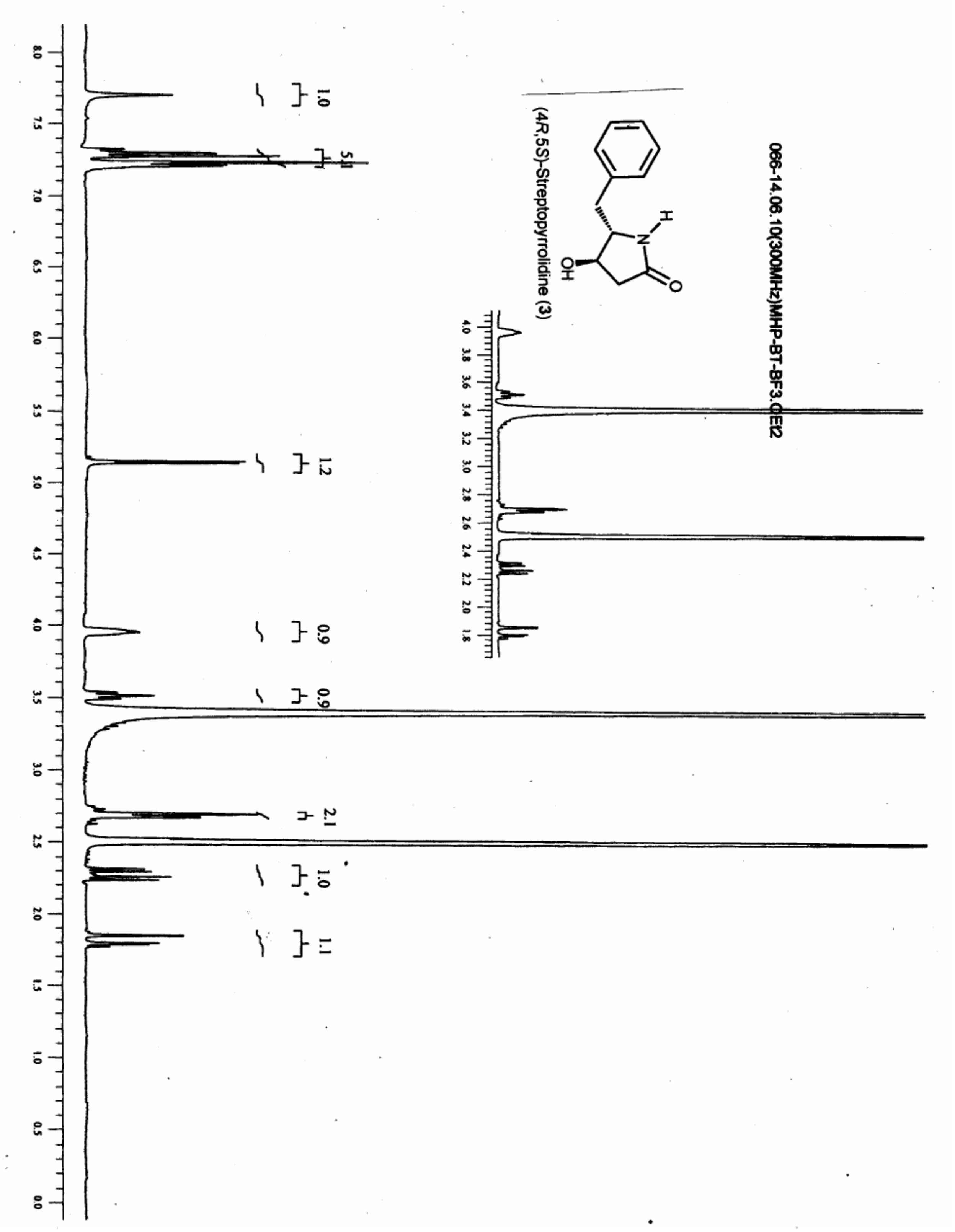
066-14.06.10(300MHz)MHP-BT-BF3.OEt2
(4R,5S)-Streptopyrrolidine (3)
1.0
5.1
1.2
0.9
0.9
2.1
1.0
1.1
8.0
7.5
7.0
6.5
6.0
5.5
5.0
4.5
4.0
3.5
3.0
2.5
2.0
1.5
1.0
0.5
0.0

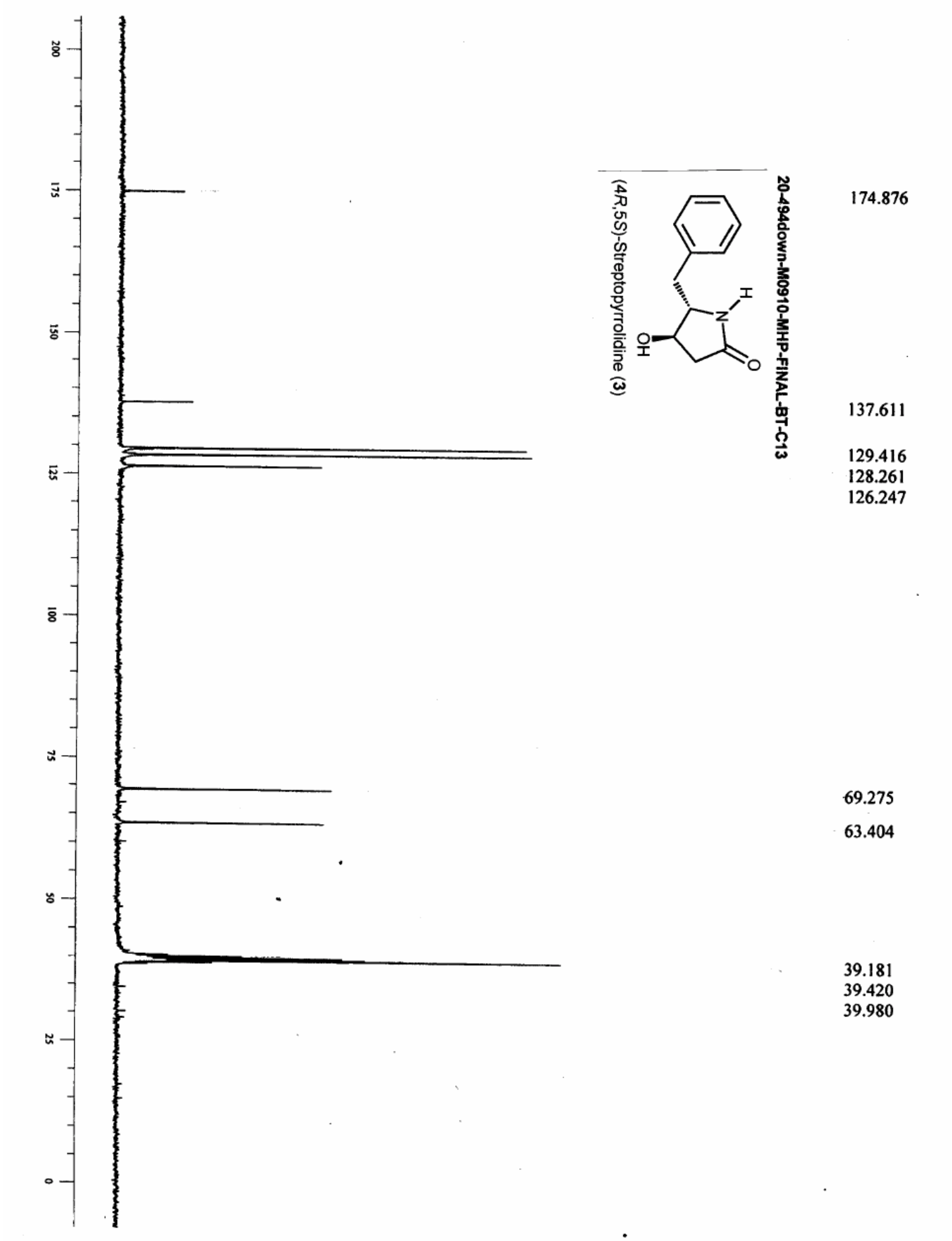

20-494down-M0910-MHP-FINAL-BT-C13
(4R,5S)-Streptopyrrolidine (3)
174.876
137.611
129.416
128.261
126.247
69.275
63.404
39.181
39.420
39.980
200
175
150
125
100
75
50
25
0

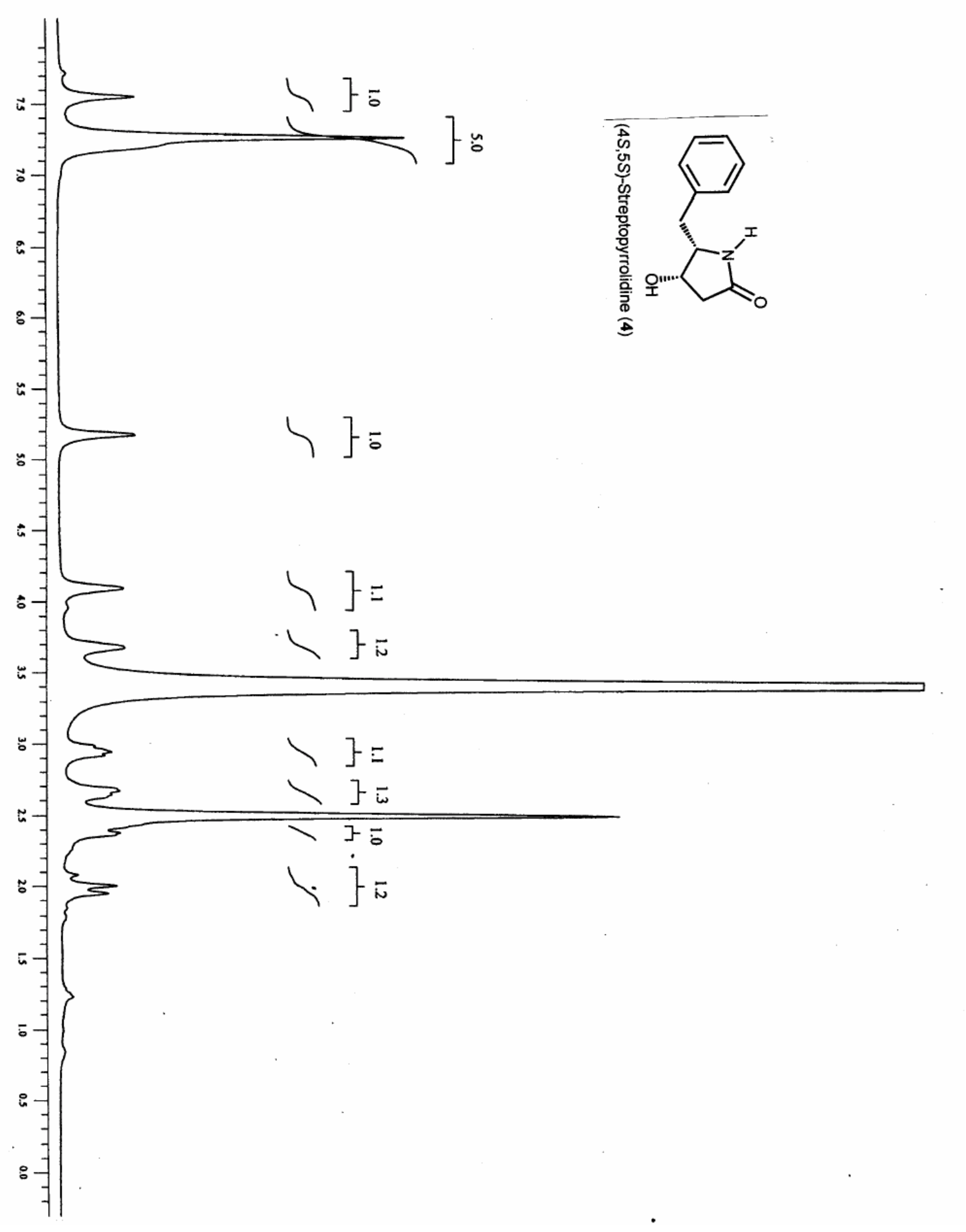
(4S,5S)-Streptopyrrolidine (4)
OH
H
N
O
5.0
1.0
1.0
1.1
1.2
1.1
1.3
1.0
1.2
7.5
7.0
6.5
6.0
5.5
5.0
4.5
4.0
3.5
3.0
2.5
2.0
1.5
1.0
0.5
0.0

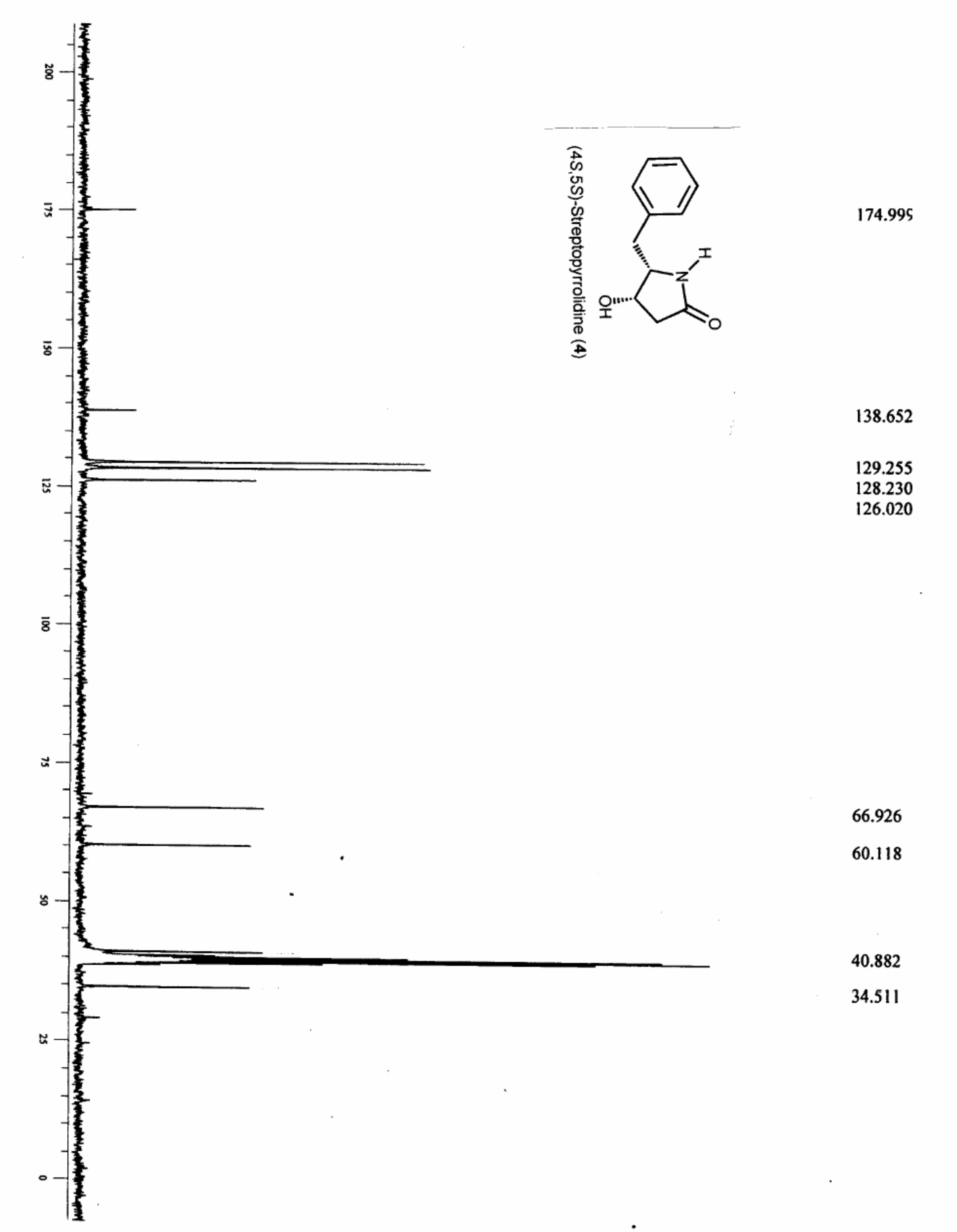

(4S,5S)-Streptopyrrolidine (4)
174.999
138.652
129.255
128.230
126.020
66.926
60.118
40.882
34.511
200
175
150
125
100
75
50
25
0
